# Tumor-Derived Exosomes Regulate Apoptosis of CD45^+^EpCAM^+^ Cells in Lung Cancer

**DOI:** 10.3389/fimmu.2022.903882

**Published:** 2022-05-30

**Authors:** Shixiang Lu, Zhen Sun, Lili Liu, Peng Li, Bin Li, Wenjing Li, Zhaojun Wu, Mingming Zhao, Wenna Liu, Yongjie Wang, Bin Wang

**Affiliations:** ^1^Department of Special Medicine, School of Basic Medicine, Qingdao University, Qingdao, China; ^2^Department of Research and Development, Sino-Cell Biomed Co., Ltd., Qingdao, China; ^3^School of Basic Medicine, Qingdao University, Qingdao, China; ^4^Department of Thoracic Surgery, The Affiliated Hospital of Qingdao University, Qingdao, China

**Keywords:** CD45^+^EpCAM^+^ cells, HCC827 cells, tumor-derived exosomes, PBMC, apoptosis

## Abstract

Lung cancer has the highest mortality rate among human cancers, and the majority of deaths result from metastatic spread. The tumor microenvironment plays an important role in suppressing the immune surveillance and elimination of tumor cells. A few studies have reported the presence of CD45^+^EpCAM^+^ double-positive cells in cancer, but the underlying mechanism remains unclear with respect to how these cells originate and their function in cancer biology. In this study, we analyzed 25 lung tumor samples. We confirmed the presence of CD45^+^EpCAM^+^ cells in lung cancer, and these cells exhibited higher apoptosis than CD45^+^EpCAM^−^ cells. Using co-culture of lung cancer cell-derived exosomes with healthy donor peripheral blood mononuclear cells, we recapitulated CD45^+^EpCAM^+^ cell formation and increased apoptosis that occurs in patients with primary lung cancer. Further analysis suggested that microRNAs in lung cancer cell-derived exosomes may alter the gene expression profile of CD45^+^EpCAM^+^ cells, resulting in elevated *TP53* expression and increased apoptosis. To our knowledge, this is the first report of cancer cell-derived exosomes that can inhibit the immune system by promoting immune cell apoptosis.

## Introduction

Lung cancer is one of the leading causes of cancer-related deaths in both men and women (1) and remains the most commonly diagnosed cancer in the world (2). It is divided into two histological subtypes: non-small cell lung cancer (NSCLC), which accounts for 85% of the cases, and small cell lung cancer, which accounts for the remaining 15% (3). Surgical treatment, chemotherapy, radiotherapy, and targeted therapy are currently the most effective modalities for treating lung cancer. Unfortunately, the outcome for lung cancer patients remains unsatisfactory. Many studies have indicated that the chemical resistance and highly metastatic nature of epithelial tumors are associated with epithelial-mesenchymal transformation (EMT) (4, 5). Ishizawa et al. demonstrated that CD45^+^EpCAM^+^ cells exist in both solid cancer tissues and malignant pleural effusions in patients with NSCLC (6). The CD45^+^EpCAM^+^ cell population was found in three patients with EGFR mutation, and this cell population was highly suspected of exhibiting the EMT phenotype (6). Moreover, CD45^+^EpCAM^+^ cells are not only less sensitive to standard drug regimens but also more invasive and equipped to avoid natural killer (NK) cell-mediated immune surveillance in human epithelial ovarian carcinoma (EOC) (7). However, there are few studies regarding the manner in which CD45^+^EpCAM^+^ cells are formed.

Exosomes are secreted by almost all types of cancer cells; these are extracellular vesicles, 30–150 nm in diameter (8). Nucleic acids (9), proteins (10), and lipids (11) are delivered to neighboring or remote cells and modulate recipient cells by cancer-derived exosomes. Recently, high levels of microRNAs (miRNAs) have been identified in cancer-derived exosomes, which provide an advantageous microenvironment for promoting tumorigenesis (12), tumor metastasis (9, 10), angiogenesis (13), chemoresistance (14), and immune escape (15). For example, lung cancer cell‐derived exosomes that upregulate miR-28-5p, were shown to facilitate mesenchymal stem cell function in phosgene-induced acute lung injury (16).

An increasingly number of exosomes are identified as a mode of long-distance intercellular communication from sites in tissues to the circulation (17, 18). When released into the extracellular milieu, exosomes communicate *via* signals by intercellular shuttling that transports macromolecules, to promote tumor growth and immunological tolerance, locally and systemically (19–21). Acting as cellular substitutions, exosomes are important contributors to the damnification of the immune system (22). Although the role of exosomes in attenuating immunoreaction is not well known, recent research indicates that immune cells can be guided toward a tumor-promoting phenotype by cancer-derived exosomes and facilitate tumorigenesis, intrusion of the peripheral tissues, angiogenesis, formation of pre-metastatic niches, and metastatic dissemination (23). Several investigations indicate that exosomes from tumor cells possess dissimilar immunosuppressive effects, including the inhibition of effector T cell activity (24), differentiation of fibroblasts to a myofibroblastic phenotype (25), and the functional enhancement of regulatory T cells.

In this study, we report the presence of CD45^+^EpCAM ^+^ cells in NSCLC tumor tissue, and these cells are prone to undergoing apoptosis. Co-culture of exosomes derived from HCC827 human lung cancer cells with PBMCs resulted in the formation of CD45^+^EpCAM^+^ cells. Further studies showed that miRNA from exosomes may play a role in changing the gene expression profiles of CD45^+^EpCAM^+^ cells to impair their antitumor activity. The p53 pathway may be one of the targets of the miRNA in exosomes, which renders these cells susceptible to apoptosis. Our data reveal a new potential mechanism of how tumor cells inhibit the immune system by producing exosomes that deliver molecules to alter immune cell function.

## Materials and Methods

### Human Lung Cancer Cells

Twenty-five patients with lung cancer were recruited from a local hospital in Qingdao, China, between 2021 and 2022. This research was approved by the Ethics Committee of the Medical College of Qingdao University, under the accession number QDU-HEC-2022157. The patients were histopathologically diagnosed by at least two pathologists, according to the World Health Organization classification. No history of cancer and any antitumor therapy occurred prior to the primary diagnosis. Fresh tumor specimens were acquired using minimally invasive surgery followed by single-cell preparation as described further. Fresh lung tumor specimens were cut into small pieces of about 1–3 mm, followed by the addition of an appropriate amount of RPMI-1640 medium (CM31800; G-Clone) containing 10% FBS (SV30087.02; HyClone) on a 40-mm cell strainer (352340; FALCON) and gentle trituration with a 20-ml syringe plunger until homogeneous cell suspensions were acquired. Subsequently, the suspended cells were filtered with cell strainers followed by centrifugation at 400*g* for 10 min. Finally, the cell pellets were resuspended in RPMI-1640 medium with 10% FBS after washing twice using 1× PBS.

### PBMCs Extraction and Culture

PBMCs were isolated from healthy donors using Density Reagent (DAKEWE). After density gradient centrifugation for 30 min at 700*g*, the PBMCs that settled at the interphase were carefully collected and washed twice with 1× PBS. PBMCs were cultivated in RPMI-1640 medium replenished with 10% human AB serum (Gemcell), 1% penicillin and 1% streptomycin (Gibco), CD3 (50 ng/ml; Beijing T&L Biotechnology), and Interleukin-2 (300 U/ml; Kingsley). PBMCs were conventionally sustained in a humidified incubator at 37°C with 5% CO_2_, and the culture medium was substituted every other day.

### Cell Culture of HCC827 Cells

The HCC827 human lung cancer cell line was acquired from the American Type Culture Collection and cultivated in RPMI-1640 medium supplemented with 10% exosome-free fetal bovine serum (C3801-0100; VivaCell), 1% penicillin and 1% streptomycin. HCC827 cells were conventionally sustained in a humidified incubator at 37°C with 5% CO_2_, and the culture medium was substituted every second day. HCC827 cells were secondary cultured until they reached 80%–90% confluence.

### Flow Cytometry and Cell Sorting

Cells for surface staining were obtained from the tumors or peripheral blood. The following fluorochrome-conjugated antibodies were utilized: anti-human CD19 (4G7), anti-human CD4 (OKT4), anti-human CD8 (SK1), anti-human CD3 (OKT3), anti-human EpCAM (9C4), anti-human CD45 (2D1), anti-human PD-1 (A17188B), and anti-human CD69 (FN50) (all from BioLegend). Flow cytometry data was acquired from a CytoFLEX (Beckman-Coulter, Fullerton, CA, USA) and analyzed by FlowJo software (TreeStar). The surface-stained cells were sorted using a FACSAria II (BD Biosciences) to reach more than 90% purity.

### Analysis of Apoptosis

After cell surface staining, apoptosis assays were performed by staining cells with Annexin V detection kit (559763; BD Pharmingen). Flow cytometry data was collected from a CytoFLEX and analyzed by FlowJo software.

### Immunofluorescent Histochemical Staining

Tumor samples from patients with lung cancer were frozen with OCT (Sakura Finetek) in liquid nitrogen. Cryostat Microtome System was used for cutting tissues into 8-μm thick sections. The tissue sections were fixed in 4% paraformaldehyde (P0099; Beyotime) for 30 min and permeabilized by incubating in PBST (0.2% Triton X-100 in PBS) (T8787; Sigma-Aldrich) at 25°C for 20 min. The samples were blocked for 1 h at room temperature in PBS with 5% bovine serum albumin (A8010; Solarbio), and 0.05% Tween 20 (P9416; Sigma-Aldrich). The sections were then incubated with anti-EpCAM antibody (ab71916; Abcam; 1:100). Subsequently, Cy3-conjugated goat anti-rabbit IgG (GB21303; Servicebio; 1:300) and FITC conjugated CD45 antibodies (ab197730; Abcam; 1:100) were used for incubating the sections. The PBS was used to wash slides at least thrice after each procedure. The sections were immobilized and mounted with an antifade kit including DAPI (P0131; Beyotime Biotechnology) and subsequently inspected with a confocal fluorescence microscope.

### Exosome Isolation, Characterization, and Treatment

As described, exosomes were obtained from cell culture supernatants by differential centrifugation (26). HCC827 cells were cultured in RPMI-1640 medium using exosome-free serum. After the supernatant was collected, centrifugation at 300g for 10 min at 4°C was used for the removal of non-adherent cells. The second centrifugation at 2,000g for 10 min at 4°C was followed by a third one at 10,000g for 30 min at 4°C, with the supernatant being transferred to a clean tube during each round. Finally, the exosomes were pelleted *via* ultracentrifugation at 120,000g for 70 min at 4°C. Subsequently, the exosome pellet was washed with 1× PBS and centrifuged at 120,000g for another 70 min, followed by resuspension in 1× PBS. The BCA protein assay kit (PC0020; Solarbio) was used to measure the exosome concentration. The exosome characterization was ascertained by HT7700 (Hitachi, Japan) TEM and exosome size by N30E Nanoparticle Tracking analysis (NanoFCM, China). CD63^+^ human exosomes were analyzed from a pre-enriched exosome solution prepared using ultracentrifugation for flow cytometry analysis using the Human CD63 Isolation/Detection kit (10606D; Invitrogen). Purified HCC827 cell-derived exosomes were used for experimental procedures. For *in vitro* experiments, PBMCs from healthy donors were incubated with exosomes for 24 h (10, 50, and 100 µg/ml).

### RNA Isolation, Reverse Transcription, and Quantitative RT-PCR

Total RNA was extracted using the RNAfast200 kit (Fastagen, China). RNA was reverse-transcribed using the HiScript^®^ III RT SuperMix (Vazyme, China). Relative gene expression levels were analyzed by quantitative RT-PCR (qRT-PCR) with the ChamQ Universal SYBR qPCR Master Mix (Vazyme, China) on a Roche Light Cycler 480 System (Roche, Basel, Switzerland). The primer sequences were: *PTPRC*: 5′-ATA CTG GCC GTC AAT GGA AGA-3′ and 5′-CAG TTT GAG GAG CAA GTG AGG A-3′, *VIM*: 5′-CAT GAC CTC TAC GAG GAG GAG ATG C-3′ and 5′-TGT CTG AAA GAT TGC AGG GTG T-3′, *ZEB1*: 5′-AGG TGT AAG CGC AGA AAG CAG-3′ and 5′-CCT CCC AGC AGT TCT TAG CAT T-3′, *EPCAM*: 5′-ATG ATC CTG ACT GCG ATG AGA G-3′ and 5′-TGA TAA CGC GTT GTG ATC TCC T-3′, *CDH1*: 5′-CCA CCA AAG TCA CGC TGA ATA C-3′ and 5′-CTG ATG GGA GGA ATA ACC CAG T-3′, *TP53*: 5′-AGC ATC TTA TCC GAG TGG AAG G-3′ and 5′-CAG TGT GAT GAT GGT GAG GAT G-3′, and *IFNG*: 5′-TCG GTA ACT GAC TTG AAT GTC CA-3′ and 5′-TCG CTT CCC TGT TTT AGC TG C-3′, and *GAPDH*: 5′-CAT GTT CGT CAT GGG TGT GAA-3′ and 5′-CAT GGA CTG TGG TCA TGA GTC CT-3′.

### siRNA Transfection

siRNA duplexes, both siRNA-control (sc-37007) and siRNA-p53 (sc-29435), as well as siRNA transfection reagent (sc-29528) were purchased from Santa Cruz Biotech. The procedure was performed according to the manufacturer’s instructions. Briefly, 2 × 10^6^ PBMCs were plated into each well and transfected with 20 nM siRNA in a six well plates. After 6 hours, 1 ml of HCC827 culture supernatant containing 20% exosome-free fetal bovine serum, 2% penicillin and 2% streptomycin were added into each well without removing the transfection mixture followed by incubating for an additional 18-24 hours. Afterwards, PBMCs were harvested and replaced with HCC827 culture supernatant containing 10% exosome-free fetal bovine serum, 1% penicillin and 1% streptomycin. 24 hours later, apoptosis assays and RT-PCR were performed.

### Small RNA Sequencing and Data Analysis

Total RNA from HCC827 cell-derived exosomes was isolated with the exoRNeasy Maxi Kit (Qiagen). Next, the sequencing library was established using the high-quality RNA; 3 μg total RNA was used as raw material for the small RNA library. The NEBNext Multiplex Small RNA Library Prep Set for Illumina (NEB, USA) was used for establishing small RNA libraries. An Illumina platform from Novogene Corporation (Beijing, China) was used to sequence the libraries. For miRNA-seq data analysis, the raw data quality was evaluated with FastQC. Clean data was aligned to the latest miRBase20.0 database, and the remaining readings were aligned with the latest human genome.

### Statistical Analysis

Using GraphPad Prism software (version 8.0), the statistical significance of the differences between the groups was confirmed by a two-tailed, unpaired Student’s *t*-test with 95% confidence interval. Differences with *P* ≥ 0.05 were considered insignificant (NS). *P* values <0.05 were considered statistically significant (*P < 0.05; **P < 0.01; ***P < 0.001).

## Results

### CD45^+^EpCAM^+^ Cells Are Detected in Early Stages of Lung Cancer

**Figure 1:**
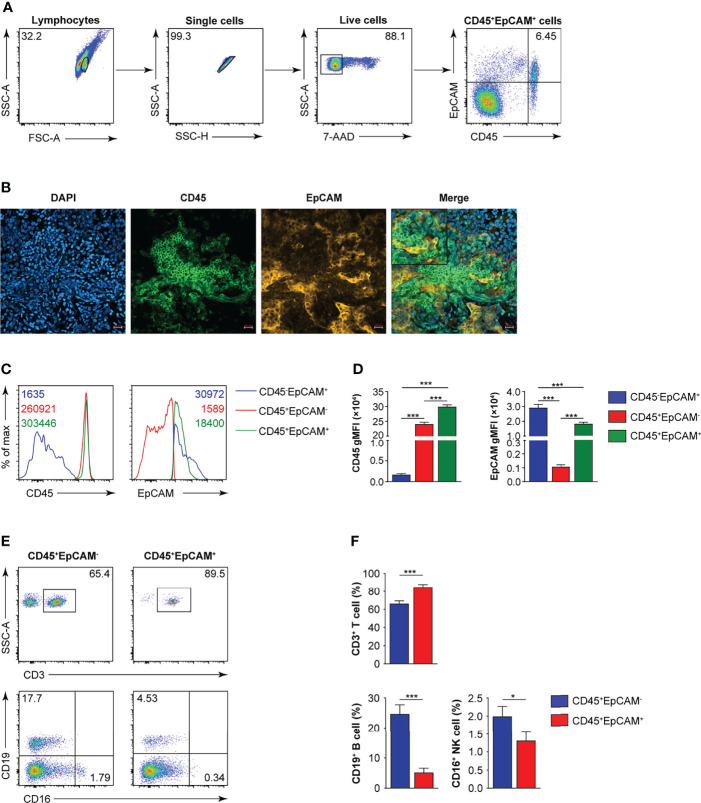
CD45^+^EpCAM^+^ cells are detected in lung cancer tissue by FACS and immune-fluorescence microscopy. **(A)** Flow cytometry analysis of CD45^+^EpCAM^+^ cells gated on live cells from solid tumors of patients with lung cancer with representative pseudocolor plot, respectively (average of CD45^+^EpCAM^+^ in lung cancer is 6.25%, *n =* 25). **(B)** Confocal microscopy analysis of CD45^+^EpCAM^+^ cells in solid tumors from patients with lung cancer. Green: CD45, red: EpCAM. Scale bar: 20 μm. Representative figures are presented from two independent experiments. **(C, D)** Expression of CD45 and EpCAM on CD45^+^EpCAM^+^ cells was analyzed using flow cytometry with typical histograms and quantification data in **(C, D)**, respectively (*n =* 25). **(E, F)** Flow cytometry analysis of CD3^+^ T, CD19^+^ B, and CD16^+^ cells gated on CD45^+^EpCAM^+^ cells or CD45^+^EpCAM^−^ cells from tumors of patients with lung cancer with typical pseudocolor plots and cumulative data in **(E, F)**, respectively (*n =* 25). Data are representative of 25 separate experiments. Error bars represent SEM. *P < 0.05, and ***P < 0.001 (Student’s *t*-test).

CD45 is a marker for leukocytes, and EpCAM is a marker for epithelial cells. In 25 tumor tissues from patients with early lung cancer, we confirmed the existence of CD45^+^EpCAM^+^ cells by FACS and immunofluorescent histochemical staining (**Figures 1A, B**). The expression levels of CD45 and EpCAM were markedly higher in CD45^+^EpCAM^+^ cells than in CD45^+^EpCAM^−^ cells (**Figures 1C, D**). Next, we examined the percentage of different immune cell subsets in the CD45^+^EpCAM^+^ population. Flow cytometry revealed a significantly higher percent of CD3^+^ T cells in the CD45^+^EpCAM^+^ subsets than of CD45^+^EpCAM^−^ cells (**Figures 1E, F**). In contrast, significantly fewer CD19^+^ and CD16^+^ cells in the EpCAM^+^CD45^+^ subsets were present than CD45^+^EpCAM^−^ cells (**Figures 1E, F**).

### Majority of EpCAM^+^CD45^+^ Cells Expressing PD-1 and CD69

**Figure 2:**
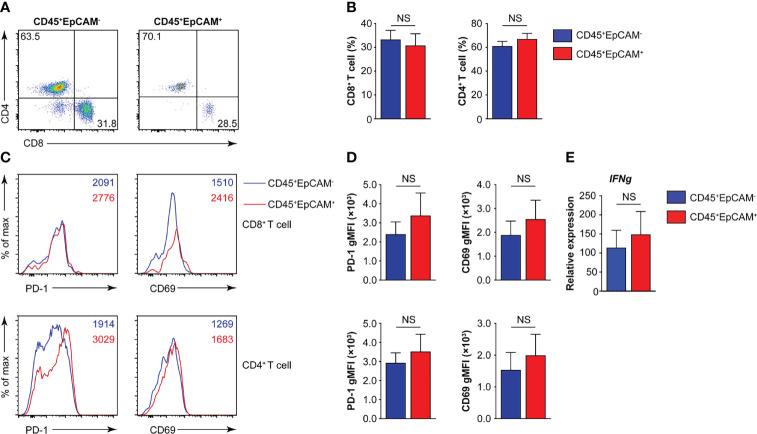
CD45^+^EpCAM^+^CD8^+^ T cells in lung cancer express PD-1 and CD69. **(A, B)** Flow cytometry analysis of CD8^+^ T and CD4^+^ T cells gated on CD45^+^EpCAM^+^CD3^+^ and CD45^+^EpCAM^−^CD3^+^ cells from tumors of patients with lung cancer with representative pseudocolor plot and cumulative data in **(A)** and **(B)**, respectively (*n =* 8). **(C, D)** Expression of PD-1 and CD69 on CD8^+^ T and CD4^+^ T cells was analyzed using flow cytometry with typical histograms and quantification data in **(C)** and **(D)**, respectively (*n* ≥ 8). **(E)** Quantitative RT-PCR analysis of *IFNg* from sorted CD45^+^EpCAM^+^ and CD45^+^EpCAM^−^ cells after PBMCs cultured with HCC827 cell conditioned media (*n* ≥ 4) NS, no significance.

The programmed death 1 receptor (PD-1), known as an immunoinhibitory receptor, is expressed by chronically stimulated CD8 T cells (27–29). These PD-1^+^ CD8 T cells demonstrate a reduced proliferation capacity and express effector cytokines. CD69 is known as an activation marker and is expressed on infiltrated leukocytes at inflammatory sites under various chronic human inflammatory diseases, such as rheumatoid arthritis (30), systemic sclerosis (31), allergic asthma (32), and atopic dermatitis (33). Therefore, we first analyzed the proportion of CD8^+^ and CD4^+^ T cells associated with CD45^+^EpCAM^+^ cells. The results indicated that there was no difference in the ratio of CD4^+^ T to CD8^+^ T cells in CD45^+^EpCAM^+^ cells compared with CD45^+^EpCAM^−^ cells (**Figures 2A, B**). Moreover, there was no difference in PD-1 and CD69 expression in CD8^+^ T and CD4^+^ T cells between CD45^+^EpCAM^+^ and CD45^+^EpCAM^−^ cells (**Figures 2C, D**). There was also no considerable difference in IFNγ production between CD45^+^EpCAM^+^ and CD45^+^EpCAM^−^ cells (**Figure 2E**). These results indicated that CD45^+^EpCAM^+^ T cells display an effective antitumor immune response.

### CD45^+^EpCAM^+^ Cells Display Elevated Apoptosis

**Figure 3:**
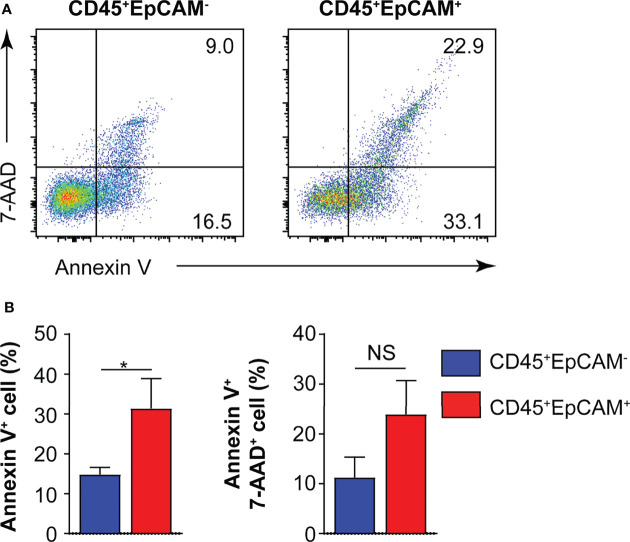
CD45^+^EpCAM^+^ cells undergo apoptosis in the tumor microenvironment. **(A)** CD45^+^EpCAM^+^ and CD45^+^EpCAM^−^ cells from tumors of patients with lung cancer were stained with Annexin V/7-AAD kit to assess viability by flow cytometry. **(B)** The ratio of apoptosis in CD45^+^EpCAM^+^ and CD45^+^EpCAM^−^ cells is shown in **(B)**, respectively (*n =* 6). Data are representative of six separate experiments. Error bars represent SEM. *P < 0.05 (Student’s *t*-test). NS, no significance.

To determine whether CD45^+^EpCAM^+^ cells are different from CD45^+^EpCAM^−^ cell with respect to viability, we used Annexin V staining to quantitate apoptosis levels. We found a significantly higher rate of apoptotic cells in the CD45^+^EpCAM^+^ versus CD45^+^EpCAM^−^ cell population (**Figures 3A, B**). These data suggest that increased apoptosis of CD45^+^EpCAM^+^ cells may contribute to immune suppression in lung cancer.

### HCC827 Lung Cancer Cell-Derived Exosomes Fuse With Healthy Donor PBMCs to Form CD45^+^EpCAM^+^ Cells

There are several potential mechanisms of CD45^+^EpCAM^+^ cell formation in cancer tissue. One possibility is the direct contact of cancer cells (expressing EpCAM) with CD45^+^ blood cells, which may result in fusion to form CD45^+^EpCAM^+^ cells. The second possibility is that exosomes derived from cancer cells (or blood cells) fuse with blood (or cancer) cells. The majority of the CD45^+^EpCAM^+^ cells from the FACS analysis are in a single-cell gate, which suggests that the cells are unlikely to result from cell–cell fusion. Thus, we focused on the second possibility, which is to determine whether exosomes derived from cancer cells (or blood cells) induce CD45^+^EpCAM^+^ cell formation. We first added conditioned media from human lung cancer cell line HCC827 to peripheral blood mononuclear cells (PBMCs) and did the same vice versa. We observed a significant level of CD45^+^EpCAM^+^ cell formation when conditional media from HCC827 was added to the PBMCs culture system. In contrast, there were few CD45^+^EpCAM^+^ cells present when PBMCs conditional media was added to HCC827 cells (data not shown). The cell growth rate was not affected in either experiment indicating that conditioned media did not affect cell growth.

**Figure 4:**
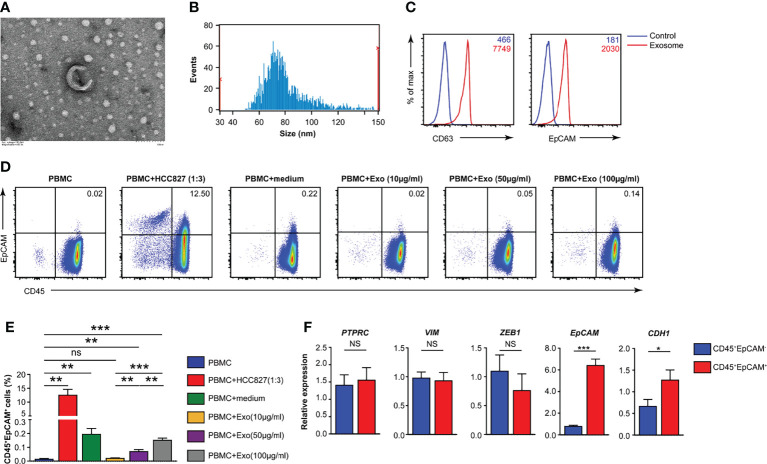
HCC827 cell-derived exosomes play a critical role in CD45^+^EpCAM^+^ cell formation. **(A)** Representative TEM pictures of HCC827 cell-derived exosomes. Scale bar: 100 nm. **(B)** Nanoparticle tracking the size distribution of HCC827 cell-derived exosomes. **(C)** Detection of protein levels of CD63 and EpCAM in HCC827 cell-derived exosomes by flow cytometry (representative of 3 independent experiments). **(D, E)** Flow cytometry analysis of CD45^+^EpCAM^+^ cells from PBMCs cocultured with HCC827 cells, cultured with HCC827 cell media, or with HCC827 cell-derived exosomes (10, 50, and 100 µg/ml) for 24 h. Analysis of CD45^+^EpCAM^+^ and CD45^+^EpCAM^−^ cells was from the same sample, and 5 × 10^5^ live cells were analyzed for each sample. Representative pseudocolor plot and cumulative data are demonstrated in **(D, E)**, respectively (*n* = 3). **(F)** Quantitative RT-PCR analysis of epithelial and mesenchymal marker genes described as above in CD45^+^EpCAM^+^ and CD45^+^EpCAM^−^ cells from PBMCs cultured with HCC827 cell conditioned media (*n* ≥ 4). Data are representative of at the latest three separate experiments. Error bars delegate SEM. *P < 0.05, **P < 0.01, and ***P < 0.001 (Student’s *t*-test). NS, no significance.

To identify the components in the conditioned media of the HCC827 human lung cancer cell line that mediated this phenotypic switch of CD45^+^ immune cells, we determined whether the exosomes were a significant contributor. Exosomes were isolated from the culture supernatants of HCC827 cells through multiple rounds of centrifugation. Using transmission electron microscopy (TEM), the HCC827-derived exosomes were perceived to be circular vesicles (**Figure 4A**), and exosome sizes in the range of 30–150 nm were detected by nanoparticle tracking analysis (**Figure 4B**). Flow cytometry revealed that CD63 and EpCAM were co-expressed in the isolated exosomes (**Figure 4C**). We isolated PBMCs from healthy donors and cocultured them under various conditions (**Figure 4D**). We observed CD45^+^EpCAM^+^ cells when PBMCs were cocultured with HCC827, with HCC827 conditioned media, and with different amounts of purified exosomes (**Figures 4D, E**). The formation of CD45^+^EpCAM^+^ cells in PBMCs/exosome co-culture occurred in an exosome dose-dependent manner. Next, we sorted CD45^+^EpCAM^+^ and CD45^+^EpCAM^−^ cells to examine gene expression. We found that the expression of the epithelial cell-related genes, *EPCAM* and *CDH1*, was markedly increased in CD45^+^EpCAM^+^ cells by quantitative RT-PCR analysis (**Figure 4F**). In contrast, the expression of mesenchymal cell-relevant genes *PTPRC*, *VIM*, and *ZEB1* did not show significant differences in CD45^+^EpCAM^+^ cells compared with CD45^+^EpCAM^−^ cells (**Figure 4F**).

### CD45^+^EpCAM^+^ Cells From PBMCs and Exosome Co-Culture Show Increased Apoptosis

**Figure 5:**
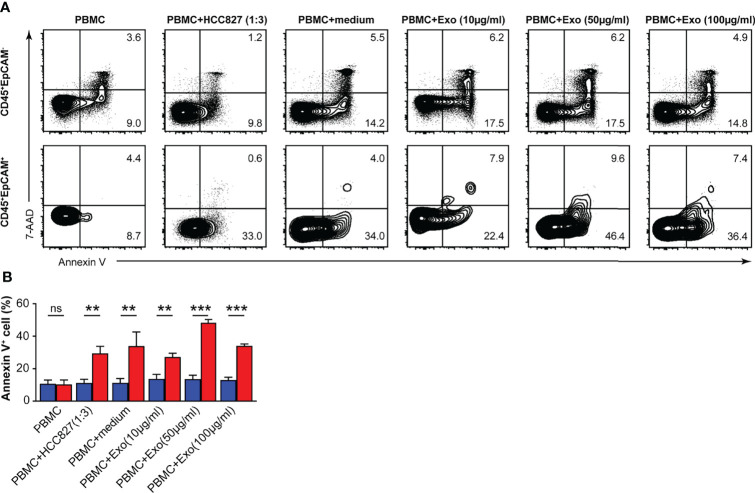
HCC827 cell-derived exosomes induce CD45^+^EpCAM^+^ cells from PBMCs that are prone to apoptosis. **(A)** Contour plots represent Annexin V^+^ cells gated on CD45^+^EpCAM^−^ (top panel) and CD45^+^EpCAM^+^ cells (bottom panel) from PBMCs, cocultured with HCC827 cells, or with HCC827 cell media, or with different amounts of HCC827 cell-derived exosomes (10, 50, and 100 µg/ml) for 24 h. Data are representative of three separate experiments. **(B)** Quantification data on the ratio of Annexin V^+^ cells are demonstrated (*n* = 3). Error bars represent SEM. **P < 0.01, and ***P < 0.001 (Student’s *t*-test). NS, no significance.

We measured the apoptosis rate of CD45^+^EpCAM^+^ and CD45^+^EpCAM^−^ cells in PBMCs after HCC827 cell-derived exosome treatment. We found that the apoptosis ratio was increased in CD45^+^EpCAM^+^ cells compared with CD45^+^EpCAM^−^ cells from PBMCs co-cultured with HCC827 cells, HCC827 media, and different amounts of exosomes (**Figures 5A, B**). Moreover, apoptosis was increased in CD45^+^EpCAM^+^ cells when PBMCs were co-cultured with higher amounts of HCC827-derived exosomes.

### HCC827 Cell-Derived Exosomes Promote CD45^+^EpCAM^+^ Cell Apoptosis *via* the p53 Pathway

**Figure 6:**
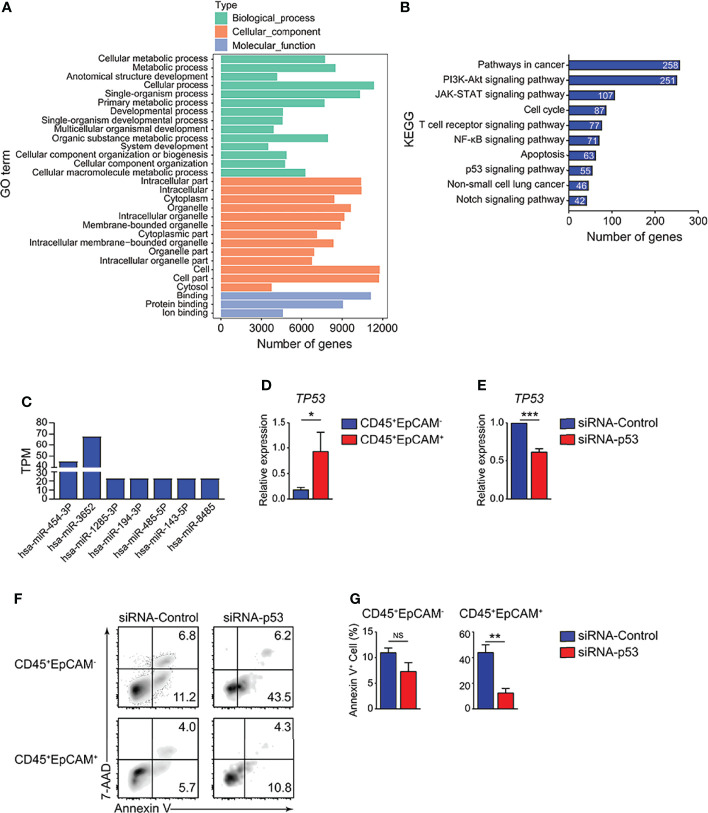
HCC827 cell-derived exosomes promote CD45^+^EpCAM^+^ cell apoptosis *via* the p53 pathway. **(A)** GO function analysis for miRNA-targeted genes in HCC827 cell-derived exosomes. **(B)** KEGG pathway analysis of miRNA-targeted genes in HCC827 cell-derived exosomes. **(C)** Expression of miRNAs targeting gene *TP53* in HCC827 cell-derived exosomes. **(D)** Quantitative RT-PCR analysis of the cell apoptosis-related gene, *TP53*, in CD45^+^EpCAM^+^ and CD45^+^EpCAM^−^ cells from PBMCs cultured with HCC827 cell conditioned media (*n* ≥ 4). **(E)** Quantitative RT-PCR analysis of *TP53* in siRNA-control group and siRNA-p53 group from PBMCs cultured with HCC827 cell conditioned media (*n* = 3). **(F, G)** Flow cytometry analysis of Annexin V^+^ cells gated on CD45^+^EpCAM^−^ (top panel) and CD45^+^EpCAM^+^ cells (bottom panel) from PBMCs cultured with HCC827 cell conditioned media, treated with siRNA-control or siRNA-p53 treatment respectively. Data are presented by typical density plots and quantification data shown in **(F, G)**, (*n =* 3). Data are representative of at any rate two separate experiments. Error bars delegate SEM. *P < 0.05, and **P < 0.01, and ***P < 0.001 (Student’s *t*-test). NS, no significance.

To identify the mechanisms of HCC827 cell-derived exosomes in regulating CD45^+^EpCAM^+^ cell apoptosis, we analyzed the miRNA profiles of the exosomes. The miRNA data analysis revealed that numerous miRNAs were encapsulated within the exosomes (**Table S1**). GO and KEGG pathway enrichment analyses were performed for miRNA-targeted genes in HCC827 cell-derived exosomes, and the results are presented in **Figures 6A, B**. Based on the results, miRNA target genes were enriched in multiple GO categories including cellular process, metabolic process, cell organelle, and so on (**Figure 6A**). Simultaneously, miRNA-targeted genes were enriched in several signaling pathways, including pathways in cancer, PI3K-Akt signaling, JAK-STAT signaling, apoptosis, NSCLC, and particularly the p53 signaling pathway (**Figure 6B**). The miRNAs targeting genes of the p53 signaling pathway were detected in HCC827 cell-derived exosomes (**Figure 6C**). Using quantitative RT-PCR, we confirmed the *TP53* gene alterations. We found increased expression of the apoptotic-related gene, *TP53*, in CD45^+^EpCAM^+^ cells compared with CD45^+^EpCAM^−^ cells (**Figure 6D**). To further confirm the regulatory role of p53 signaling pathway in CD45^+^EpCAM^+^ cell apoptosis, we used siRNA-p53 to knock down *TP53* gene in the PBMCs-exosome co-culture system (**Figure 6E**). In CD45^+^EpCAM^+^ cell population, we found significantly decreased apoptosis in siRNA-p53 treated group compared with siRNA-control treated group (**Figures 6F, G**). Overall, these results suggest that HCC827 cell-derived exosomal miRNAs induce CD45^+^EpCAM^+^ cell apoptosis *via* the p53 pathway. However, since miRNAs can regulate multiple apoptosis-related signaling pathways, there may be other regulatory mechanisms that require further investigation.

## Discussion

Previous studies showed that CD45^+^EpCAM^+^ cells are associated with the EMT. CD45^+^EpCAM^+^ cells display the main tumor burden and more drug-resistance than EpCAM^+^ tumor cells in patients with NSCLC and EOC (6, 7). In this study, we showed that varying percentages of CD45^+^EpCAM^+^ cells exist in all of the 25 patient lung tumor tissues examined (**Figure 1**). The majority CD45^+^EpCAM^+^ cells are activated CD3^+^ T cells expressing both PD-1 and CD69 (**Figure 2**). Interestingly, our data have shown that CD45^+^EpCAM^+^ cells display elevated levels of apoptosis (**Figure 3**). This data suggest it may be another mechanism by which tumor cells suppress immune cell activity.

Latest research demonstrates that the exosome-mediated cellular material exchange between cells is a significant method of intercellular communication (34–36). Tumor-derived exosomes regulate intercellular communication and signaling pathways that influence cancer progression by transferring nucleic acids and proteins between varieties of cell genres. The latest evidence indicates that tumor-derived exosomes can also regulate immunoreaction (37). For instance, tumor-derived exosomes can be efficiently taken up by dendritic cells (DCs), and the antigen is processed and cross-presented to tumor-specific CTLs (38, 39). Du et al. revealed that LLC-derived exosomes are taken up by immune cells in the lung (40). In the present study, CD45^+^EpCAM^+^ cells were observed when PBMCs were co-cultured with HCC827, HCC827 conditioned media, and different amounts of purified exosomes. Moreover, the formation of CD45^+^EpCAM^+^ cells under PBMCs/exosome co-culture conditions occurs in an exosome dose-dependent manner (**Figures 4D, E**). We also observed CD45^+^EpCAM^+^ cell formation when the media after ultracentrifugation were used for PBMCs co-culture, although at less level compared with the purified exosomes (data not show) or media before ultracentrifugation. This suggests that ultracentrifugation did not recover all the exosomes in HCC827 conditioned media. Other components in HCC827 conditioned media may also exist and play roles to enhance the CD45^+^EpCAM^+^ cell formation to assist exosome or independently. In tumor environment, cellular mechanisms may also be involved in CD45^+^EpCAM^+^ cell formation such as recently reported “ trogocytosis” when immune cells steal tumor cell membranes carrying surface protein (41). The expression of the epithelial cell-related genes, *EPCAM* and *CDH1*, was markedly increased in CD45^+^EpCAM^+^ cells as determined by quantitative RT-PCR analysis (**Figure 4F**). These results indicate that tumor cells may influence the gene expression of immune cells through exosomes.

An increasing number of evidence demonstrates that exosomes contribute to tumor progression by transmitting immunosuppressive molecules (42). Exosomal miRNAs are vital carriers that can affect the function of immune cells containing DCs and T cells in cancer (43). Lung tumor cell-derived exosomes can transfer miR-21/29a to activate TLR7 and TLR8 in immune cells, which were promoted to tumor development and metastasis (44). In addition, the study demonstrated that tumor cell-derived exosomes can deliver miR-214 to CD4 T cells in human cancers, which ultimately reduced phosphatase and tensin homolog production and accelerated Treg cell expansion and tumor growth (45). In our study, we found a significantly higher rate of apoptotic cells in CD45^+^EpCAM^+^ versus CD45^+^EpCAM^−^ cells from solid tumors of patients with lung cancer (**Figure 3**). We recapitulated these results by co-culturing PBMCs with HCC827 cell-derived exosomes. CD45^+^EpCAM^+^ cells formed from co-culture of PBMCs with HCC827 cell derived exosomes showed increased apoptosis (**Figure 5**).

In the current research, miRNA sequencing analysis was used to confirm the functional miRNAs encapsulated in tumor derived exosomes that contribute to elevated CD45^+^EpCAM^+^ cell apoptosis. The miRNA-targeted genes were enriched in apoptotic-related signaling pathways by KEGG pathway analysis. All types of responses containing cell cycle arrest and apoptosis were generated by activated p53 (46, 47). The miRNA sequencing results indicate that the miRNAs targeting genes of the p53 signaling pathway were detected in HCC827 cell-derived exosomes, including miR454-3P, miR3652, miR1285-3P, miR194-3P, miR485-3P, miR143-3P, and miR8485 (**Figure 6C**). We confirmed that the expression of the apoptosis-related gene, *TP53*, markedly increased in CD45^+^EpCAM^+^ cells, by quantitative RT-PCR analysis (**Figure 6D**).

In summary, we demonstrated that CD45^+^EpCAM^+^ cell formation and increased apoptosis occur in patients with primary lung cancer and from PBMCs treated with HCC827 cell-derived exosomes. Further analysis suggests that miRNAs from lung cancer cell-derived exosomes may alter the gene expression profiles of CD45^+^EpCAM^+^ cells, resulting in elevated *TP53* expression and increased apoptosis. As far as we know, this research is the first to report that cancer cell-derived exosomes can inhibit the immune system by promoting immune cell apoptosis. Overall, this work disclosed a novel mechanism that is capable of inducing immune inhibition in the tumor microenvironment.

## Data Availability Statement

The datasets presented in this study can be found in online repositories. The names of the repository/repositories and accession number(s) can be found below: https://www.ncbi.nlm.nih.gov/geo/, GSE197975.

## Ethics Statement

The studies involving human participants were reviewed and approved by Approval document of Ethics Committee Medical College of Qingdao University. The patients/participants provided their written informed consent to participate in this study.

## Author Contributions

SL and ZS performed and analyzed all experiments and were involved in drafting the manuscript. LL contributed to data analysis. PL provided tumors from patients with lung cancer. ZW analyzed the miRNA-seq data. BL, WJL, MZ, and WNL assisted with the total experiments. YW and BW supervised the project and designed the experiments. All authors have read and endorsed the ultimate manuscript.

## Funding

This work was supported in part by grants from the Wu Jieping Medical Foundation to YW (320.6750.2021-01-4) and the Project for “Clinical Medicine + X” supported by the Affiliated Hospital of Qingdao University to YW (QYFY-X2021032/3731).

## Conflict of Interest

ZS, BL, WJL, ZW, MZ, and WNL are employed by Sino-Cell Biomed Co., Ltd. BW is a consultant for Sino-Cell Biomed Co., Ltd.

The remaining authors declare that the research was conducted in the absence of any commercial or financial relationships that could be construed as a potential conflict of interest.

## Publisher’s Note

All claims expressed in this article are solely those of the authors and do not necessarily represent those of their affiliated organizations, or those of the publisher, the editors and the reviewers. Any product that may be evaluated in this article, or claim that may be made by its manufacturer, is not guaranteed or endorsed by the publisher.
